# Change in microbial community in landfill refuse contaminated with antibiotics facilitates denitrification more than the increase in ARG over long-term

**DOI:** 10.1038/srep41230

**Published:** 2017-01-25

**Authors:** Dong Wu, Guanzhou Chen, Xiaojun Zhang, Kai Yang, Bing Xie

**Affiliations:** 1 Key Laboratory for Urban Ecological Processes and Eco-Restoration, School of Ecological and Environmental Science, East China Normal University, Shanghai 200241, China; 2 Joint Research Institute for New Energy and the Environment, East China Normal University and Colorado State University, Shanghai 200062, China; 3 State Key Laboratory of Microbial Metabolism, School of Life Sciences and Biotechnology, Shanghai Jiao Tong University, Shanghai 200240, China

## Abstract

In this study, the addition of sulfamethazine (SMT) to landfill refuse decreased nitrogen intermediates (e.g. N_2_O and NO) and dinitrogen (N_2_) gas fluxes to <0.5 μg-N/kg-refuse·h^−1^, while the N_2_O and N_2_ flux were at ~1.5 and 5.0 μg-N/kg-refuse·h^−1^ respectively in samples to which oxytetracycline (OTC) had been added. The ARG (antibiotic resistance gene) levels in the refuse increased tenfold after long-term exposure to antibiotics, followed by a fourfold increase in the N_2_ flux, but SMT-amended samples with the largest resistome facilitated the denitrification (the nitrogen accumulated as NO gas at ~6 μg-N/kg-refuse·h^−1^) to a lesser extent than OTC-amended samples. Further, deep sequencing results show that long-term OTC exposure partially substituted *Hyphomicrobium, Fulvivirga*, and *Caldilinea* (>5%) for the dominant bacterial hosts (*Rhodothermus*, ~20%) harboring *nosZ* and *norB* genes that significantly correlated with nitrogen emission pattern, while sulfamethazine amendment completely reduced the relative abundance of the “original inhabitants” functioning to produce NO_x_ gas reduction. The main ARG carriers (*Pseudomonas*) that were substantially enriched in the SMT group had lower levels of denitrifying functional genes, which could imply that denitrification is influenced more by bacterial dynamics than by abundance of ARGs under antibiotic pressures.

Anthropogenic processes that have more than doubled the rate of terrestrial N_2_ fixation over the past century have raised concerns of climate change, particularly stemming from the release of nitrogenated intermediates (e.g., N_2_O and NO) gas into the atmosphere^1^,^2^. This N imbalance, compounded with greenhouse gas issues, is projected to increase globally by 10% to 25% within 25 years^3^. Municipal solid waste (MSW) landfills are loaded with substantial amounts of reactive nitrogen, the mass of which accounts for 20% to 30% of the total disposed wastes^4^. Therefore, the MSW landfills with reactive nitrogen have been identified as emerging hotspots of N_2_O, with an emission flux reported to be 1 to 2 magnitudes higher than fertilized soil^5^,^6^. Microbial denitrification is considered to be a dominant pathway in reactive nitrogen removal^7^,^8^,^9^. However, this process is susceptible to interference from emerging contaminants such as heavy metals and organic toxicants, i.e., persistent organic pollutants (POPs) and antibiotics^10^,^11^,^12^. Although antibiotics are less persistent compared to metals and POPs in natural environment, some of them such as the sulfonamides (SAs) have a half-life more than 60 days and this value can extend to 300 days under dark conditions (e.g. landfills)^13^,^14^. Also, antibiotics and similar antimicrobial products are considered to be more frequent (200,000 tons/year) in household usage^15^,^16^. This level of usage has translated to higher emissions, either “intentionally” (disposal of unused or expired pharmaceuticals in the sewer or the trash bin) or “unintentionally” (excretion), and resulted in ~70% of consumed antibiotics being released to the natural environment in an active form^17^,^18^.

Recent worldwide studies show that at least 56 antibiotics belonging to six different classes were frequently detected from domestic wastes sources^19^. The antibiotics quarterly-average discharge rates of municipal wastewater treatment plants ranged from 3 to 710 g/day^20^; and each inhabitant in serving areas reportedly release 175 μg antibiotics per day^21^. According to previous research, the total antibiotics content in a MSW landfill leachate is more than 10 μg/L, slightly higher than the total content at wastewater treatment plants^22^. It has been studied that the addition of antibiotics alters the microbial community structure, and short-term exposure has a negative influence on denitrification rates^12^. However, there is strong evidence that the presence of concentrated antibiotics and lengthy incubation in a landfilling system can facilitate the propagation of antibiotic resistance genes (ARGs)^23^,^24^. It is therefore reasonable to hypothesize that the acquisition of ARGs may reduce the pressure/inhibition affected by antibiotics on denitrifying bacteria or even potentially facilitate the denitrification process because some antibiotics can be utilized as carbon sources by the resistant strains^25^. Moreover, nitrogen oxide respiration is a commonly existing physiology to increase the survival chances of microorganisms in soils^26^, and this process is facilitated by diverse microorganisms with complete or partial enzymatic machinery: nitrate (*nar* and/or *nap*), nitrite (*nir*), nitric oxide (*nor*), and nitrous oxide (*nos*) reduction. This enzymatic machinery may imply that higher bacterial diversity with denitrification redundancy could be more tolerant to antibiotics and possess higher intrinsic nitrogen metabolic capacity^27^,^28^,^29^. From this point of view, it is beneficial to explore which stage and what functional microorganisms are significantly associated with dissemination and acquisition of ARGs to rationalize N_2_ and nitrogen oxides (NO_x_) emissions in the denitrification chain.

In this study, we selected two representatives of highly concentrated antibiotics, sulfamethazine (SMT) and oxytetracycline (OTC), to discern the crosstalk of denitrification and ARG dissemination in MSW landfill refuse. Metagenomics was employed to show the variation of ARGs and denitrification gene abundance and shifts in the microbial community under different incubation conditions with the measurement of two nitrogen oxide gases (NO and N_2_O) and dinitrogen (N_2_) gas.

## Results

### Effects of antibiotics on nitrogen and nitrogen oxides gas emission

**Figure 1:**
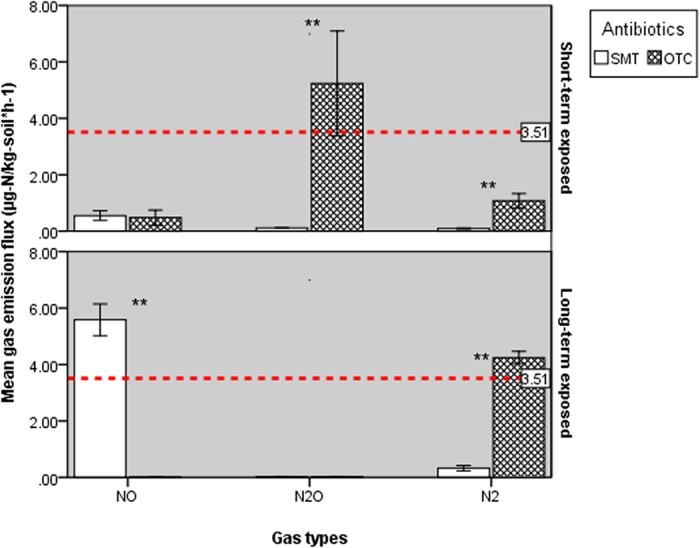
Mean nitrogen oxides (NO and N_2_O) and N_2_ gas emission flux over the period of experiments (n = 4). The error bars indicate standard deviation (±2 SD); a statistical significance (*p* < 0.01, one-way ANOVA) between the mean values was labeled with double asterisks (**). The dash line indicates the mean N_2_ flux of raw landfill refuses after the injection of leachate (without addition with antibiotics, 3.51 ± 0.61 μg-N/kg-refuse·h^−1^); the emission fluxes of NO and N_2_O from this control group were both <0.1 μg-N/kg-refuse·h^−1^).

The emissions of NO, N_2_O and N_2_ were all impacted during the period of the whole experiment (Fig. 1). The complete denitrification rate (N_2_ flux) decreased from ~3.5 μg-N/kg-refuse·h^−1^ (Fig. S1) to 1.1 and 0.07 μg-N/kg-refuse·h^−1^ (SMT) after the addition with OTC and SMT, respectively (one-way ANOVA, *p* < 0.05). Meanwhile, refuse added with OTC brought about a higher emissions flux of nitrogen intermediates where the dominant contributor was N_2_O (5.2 ± 1.8 μg-N/kg-refuse·h^−1^). However, the average emission fluxes of target gases were all below 0.5 μg-N/kg-refuse·h^−1^ in the SMT group and occurred at significantly lower rates than in the OTC-amendment group (*p* < 0.01). The intrinsic denitrification of raw samples appears to be more susceptible to SMT than to OTC. Figure 1 shows that after the long-term antibiotics incubation, N_2_ fluxes increased significantly from 1.1 ± 0.2 to 4.2 ± 0.2 μg-N/kg-refuse·h^−1^ of OTC and 0.09 ± 0.02 to 0.32 ± 0.1 μg-N/kg-refuse·h^−1^ of SMT (*p* < 0.01, paired t-test). Notably, long-term exposure of refuse to OTC-mitigated emission flux of both two target nitrogen oxide gases (paired t-test, *p* < 0.01) was essentially consistent at ~0.01 μg-N/kg-refuse·h^−1^. However, the SMT stimulated emission of NO from <0.05 to 5.6 ± 0.6 μg-N/kg-refuse·h^−1^ (paired t-test, *p* < 0.01). Denitrification could be partially streamlined with long-term incubation (compared to the short-term exposure), which can be more pronouncedly reflected in the SMT group. The total nitrogen (sum of NO, N_2_O and N_2_) gas flux is increased significantly from <0.1 to ~6.5 μg-N/kg-refuse·h^−1^ (Wilcoxon tests (WT), *p* < 0.01).

### Influence of antibiotics on the distribution of ARGs and denitrification functional genes

**Figure 2:**
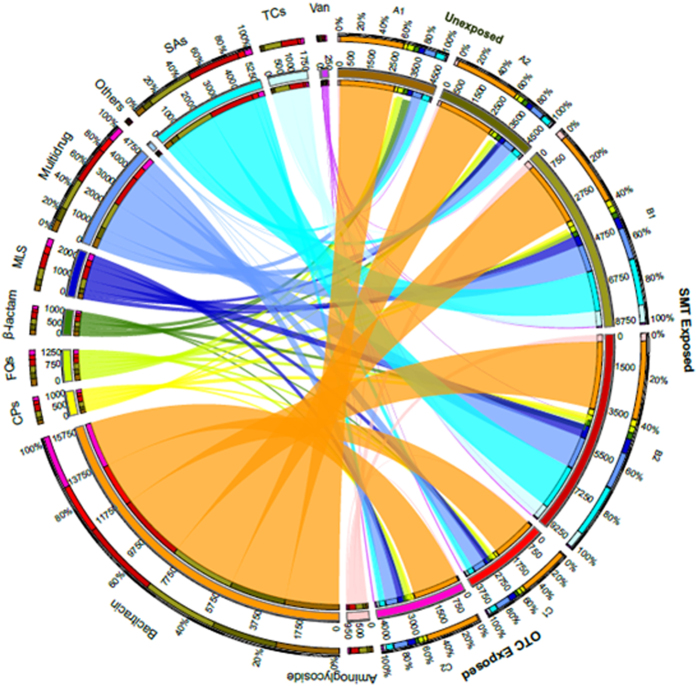
Distribution of ARG types in previously unexposed (raw refuse for short-term exposure) and exposed landfill cover refuse samples. The annotated sequences were visualized by using Circos (http://circos.ca/). The A, B and C denote previously unexposed, SMT- and OTC-amended (exposed) groups, respectively; 1 and 2 are replicate samples. The length of the bars on the outer ring represents the percentage of ARGs in each sample (right side in diagram), and the number of inner rings indicates relative abundance of annotated ARGs sequenced in each sample (ppm).

A total of 14 ARG types were detected among all samples via metagenomics sequencing, and an average of 108, 138 and 120 ARG subtypes were further assessed in the A (previously unexposed), B (OTC) and C (SMT) samples, respectively (Table S1). Figure 2 shows that the most prominent ARG types included bacitracin (44.2%), sulfonamides (SAs, 15.1%), multi-drug (13.9%), macrolide−lincosamide−streptogramin (MLS, 6.1%), tetracyclines (TCs, 5.1%), fluoroquinolones (FQs, 4.0%), chloramphenicol (CPs, 3.1%), beta-lactam (3.4%) and aminoglycoside (2.8%). Although bacitracin was not added to any samples in this experiment, the sequences (ppm) of genes coding for this type of resistance (*baca* and *bcra*) were significantly higher than any other ARGs (one-way ANOVA, n = 12, *p* < 0.05). As shown on the right side of Fig. 2, the overall ARG sequences were more distributed to long-term SMT-exposed samples (in B1 and B2, 50.9%). The percentages of *sul*^*R*^ (genes resisting to SAs) sequences increased substantially from 2.1% to 11.3%, albeit with no statistical significance detected (WT, *p* = 0.1). However, the addition of OTC imposed little impact on the abundance of *tet*^*R*^ (0.4% to 0.6%) and multidrug (2.8% to 3.2%) genes (WT, *p* = 0.55). The *tet*^*R*^ (genes resistant to TCs) sequences in the long-term OTC-exposed samples were at a significantly lower level than in the SMT groups (WT, n = 23, p < 0.05). Similarly, a higher abundance of multidrug resistance genes was detected in the SMT group compared to both OTC and unexposed groups ((WT), n = 26, p < 0.05). These results may imply that ARGs are more prone to disseminate in SMT-amended refuse versus the OTC.

All denitrification functional genes, except for *norC* and *napB*, were sequenced and annotated in this experiment (Table S2). Overall, PCA indicates broad changes in the main denitrification functional genes among the unexposed (raw refuse), SMT- and OTC-exposed samples (Fig. S2). As summarized in Table 1, the dominant genes in each denitrification step are *narG, nirS, norB* and *nosZ* with relative abundances of 0.031%, 0.021%, 0.019% and 0.016%, respectively, on average. Although long-term antibiotics incubation reduced the total abundance of genes encoding nitrate reductase (*nar* and *nap*) from ~0.07% to 0.05% and 0.04% in OTC- and SMT-amended samples, respectively, the levels of genes encoding nitrite reductases (*nirS* and *nirK*) remained at 0.004% in the OTC group. Statistically, the relative abundances of *nap, nar* and *nir* genes (functioning NO production) decreased significantly by 0.005% in the SMT group (WT; n = 12, *p* < 0.01). However, no significance was detected between unexposed and OTC-exposed groups (WT; *p* = 0.07). Interestingly, the relative abundance of *norB* was enriched in the OTC group from 0.018% to 0.23%, while the relative abundance of *norB* decreased by 0.003% in the SMT group. In contrast, the *nosZ* decreased in both of the two antibiotics-exposed samples, which was seemingly more pronounced in the SMT group from 0.02% to 0.01% on average.

### Microbial populations and denitrifying bacteria of interest in landfill refuse

Metagenomic sequencing demonstrated that 73 bacterial genera dominated (>0.1%) in one or more antibiotics-amended refuse samples. The long-term antibiotics exposure did not substantially decrease the diversity of the total bacteria, where the Shannon-Wiener index ranged from 7.04 to 7.13 (Table S3). However, the PCA results show that antibiotics exposure affected the microbial community structures substantially, particularly of potential denitrifiers (Fig. S3). Under no antibiotic stress conditions (Table S3), the predominant bacterial genera were *Sphingomonas* (6.30%), *Gemmatimonas* (3.6%), and *Ignavibacterium* (2.7%). Under the SMT- and OTC-exposed conditions, *Hyphomicrobium* (6.4%), *Anaerolinea* (5.2%) and *Pseudomonas* (1.9%) had the highest abundance. Among these genera, 20 genera of potential denitrifiers were assembled as detectable regarding carrying genes of interest (denitrification functional genes, *tet*^*R*^, *sul*^*R*^ and multi-drug resistance). Concretely, as shown in Table S4, 18 genera of potential denitrifiers were detectable (>0.1%), among which the predominant denitrifiers were *Sphingomonas* (5.8%) and *Bradyrhizobium* (1.4%). After the antibiotics amended, the main denitrifying bacteria were *Hyphomicrobium* (6.2% of SMT, 2.0% of OTC) and *Anaerolinea* (2.0% of SMT, 4.0% of OTC). Other dominant genera included *Caldilinea* (1.7%) *Nitrospira* (1.2%), *Rhodothermus* (0.7%), *Fulvivirga* (0.5%) and *Thauera* (0.3%).

## Discussion

A major finding in the present study is the contrasting three target gases that were produced during denitrification between the two antibiotics-exposure strategies. The observed results may imply that antibiotics in the landfilled refuse could inhibit denitrification in both the short- and the long-term, and their intrinsic N_2_ production capacity could decline by >50% if OTC-containing leachates were encountered. However, SMT-containing leachates may impede denitrification at an early step of N_2_O reduction. As shown in Fig. 1, this N_2_O processing (reduction) rate in SMT-exposed refuse is two orders of magnitude lower than in OTC-exposed refuse (0.09 ± 0.01 *vs*. 5.2 ± 1.8 μg-N/kg-refuse·h^−1^; t-test, *p* < 0.01) but still increased by approximately tenfold compared to the control group (Fig. S1). These results are similar to previous research results that SMT exposure reportedly elevated N_2_O flux three times to 0.07 ng-N/kg·h^−1^ 12,30,31. Apparently, the observed deficiency in denitrification of refuse may not be ascribed to a low abundance of resistance genes, given that the unexposed sample (A1 and A2) possesses substantially higher *sul*^*R*^ than *tet*^*R*^ with respect to average sequencing reads of 370 ppm and 80 ppm (Table S1).

Further, this SMT-vulnerability of landfill refuse was not apparently mended with long-term exposure to antibiotics. The increasing levels of genes encoding SMT resistance in SMT-exposed samples (Fig. 2), particularly of *sul1* (100 ppm to 750 ppm) and *sul2* (270 ppm to 1200 ppm), were inversely proportional to the NO_x_ and N_2_ gas emission matrix (Mental-test; r^2^ = 0.31, *p* < 0.01). By contrast, this emission pattern is positively correlated with the abundances of denitrifying functional gene clusters (Mental-test; r^2^ = 0.53, *p* < 0.001), where the decreased genes encoding NO reductase (*norB*, Table 1) could probably explain the increased NO flux from 0.05 to 5.6 ± 0.6 μg-N/kg-refuse·h^−1^ (paired t-test, *p* < 0.01). Interestingly, although NO_x_ reduction genes were less affected or even increased in OTC-exposed samples (e.g., *norB*), NO and N_2_O fluxes in OTC-amended samples both declined by tenfold (Fig. 1), probably because C1 and C2 steadfastly hold relatively higher levels of *nosZ* and *norB* during the whole experiment, which could lead to a higher emission of N_2_^32^,^33^. Similarly to the SMT group, analysis also indicates that the target gas emission matrix is positively correlated with denitrifying functional gene clusters (Mental-test; r^2^ = 0.84, *p* < 0.001) in OTC-exposed samples but not with *tet*^*R*^ (Mental-test; *p* > 0.05) where the dominant functional gene clusters are *tetG* (50 ppm) and *tetX* (20 ppm) on average; Table S1).

**Figure 3:**
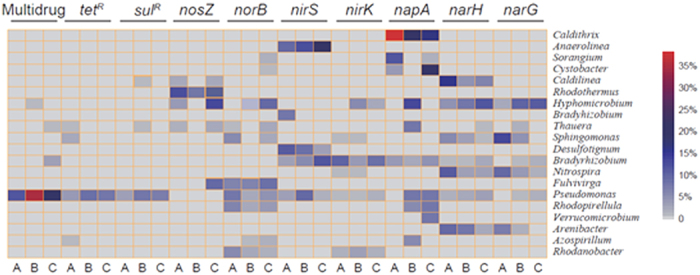
The selected denitrification functional genes were BLAST against the NCBI-nr database for taxonomic assignment (top 20 genera; gene hosts) with an e-value threshold of 10^−5^. For the reads of the 3 types of resistance genes, their taxonomic assignments were determined using BLAST against NCBI-nr, and the relative abundance of those assigned to the top 20 genera are also shown. The reads number visualized by MEGAN was taken as 100%. The A, B and C denote the mean values of two replicates of previously unexposed (raw refuse for short-term exposure), SMT- and OTC-exposed groups, respectively; and the color intensity in each panel shows the percentage of each target gene carrying a genus in one sample.

**Figure 4:**
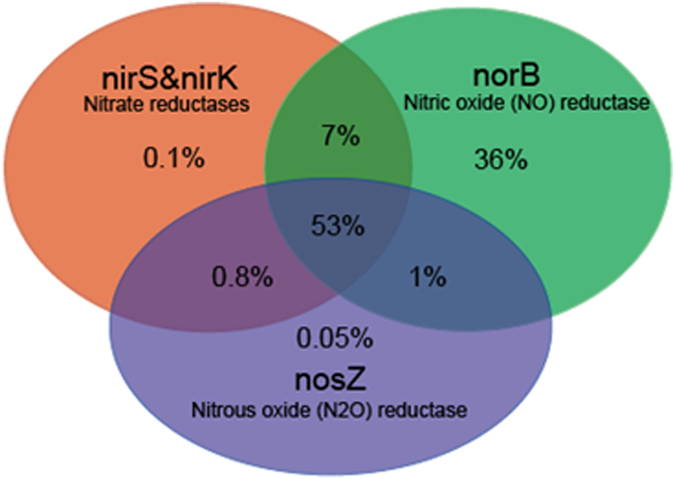
Variation partitioning analysis of target gases emission patterns explained by all denitrification functional genes, three types of ARGs, and microbial diversity by deep metagenomic sequencing; and each circle represents the variation partitioned into the relative effects of each variable or a combination of variables, in which the number indicates to what extend the emission pattern can be explained. Variables explaining more than 0.01% were illustrated in this diagram.

It is unsurprising that long-term antibiotics exposure resulted in a drastic increase of ARG abundance (Fig. 2), but the increasing trend did not correspondingly streamline the denitrification, especially under SMT-stressed conditions. According to Martinez *et al*.^34^, it is still unclear whether ARGs integrated and-or propagated via horizontal gene transfer (HGT) could confer new metabolic capabilities to the recipients. Additionally, equating the detected ARG sequences to being indicative of resistance may be biased by issues such as the existence of extracellular ARGs, which do not necessarily result in expression^35^. As shown in Fig. 3, assigned sequences regarding crosstalk of the denitrifying process and ARGs in refuse show that most potential denitrifiers are not intrinsic ARG carriers, and *Pesudomonas* was the only detectable genus owning all target genes. The VPA results show that more than half (53%) of the NO_x_ and N_2_ gas emission pattern can be explained by the variation of denitrifying functional genes, particular of *nirS*, nir*K* (0.1%) and *norB* (36%), while the target ARGs encompassing *tet*^*R*^, *sul*^*R*^ and multidrug (Fig. 4) and diversity of potential denitrifiers (Shannon-Wiener index) individually contributed <0.1% (Table S5).

The main hosts of nitrate reductase genes (*narHG* and *napA*) in unexposed raw refuse are *Caldithrix (napA*; 37.4%), *Caldilinea (narH*; 23.5%) and *Sphingomonas (narG*; 15.6%). The abundance of this microbial cluster experienced a significant decline after exposure to SMT and OTC (one-way ANOVA, *p* < 0.05). NO-producing gene (*nirS*) hosts such as *Anaerolinea* and *Bradyrhizobium* were numerically enhanced by >10% in the SMT and OTC groups, which seemingly agrees with the increasing NO flux in the SMT group (Fig. 1). However, no significant correlation was detected (Pearson correlation, r^2^ = 0.54, *p* > 0.05). It is noteworthy that the significantly lower abundances of *nap, nar* and *nir* genes in the SMT amended groups (Wilcoxon tests, *p* < 0.05, Table 1) surprisingly decreased the nitrate and nitrite (37.9 and 3.4 mg-N/kg-refuse) residuals, compared to its OTC counterparts (t-test, *p* < 0.01, Fig. S4). This may suggest that these genes and their potential functions in substrates supplies (for target NO_x_ gases), albeit negatively affected by the presence of antibiotics (Table 1), do not bottleneck the whole denitrification. Additionally, the decreased abundance of *nirK* (functioning NO production) hosts (e.g., *Rhodanobacter*, from 6.4% to <2.0% in SMT and OTC samples) and the low explanatory portion assigned to *nirS(K*) (0.1%, Fig. 4) may imply that they are not target gas emission determinants.

The hosting bacteria of potential determinant *norB* (explaining 36% of the emission pattern) in the SMT group included mainly *Hypomicrobium* (7.3%), *Fulvivirga* (10.9%) and *Rhodopirellula* (8.2%), and they were at a significantly lower level than in the OTC group (WT; n = 12, *p* < 0.01). The long-term OTC exposure substantially increased the hosts of *norB* genes functioning for NO reduction (Fig. 3) such as *Hypomicrobium* (16.5%), *Fulvivirga* (12.4%) and *Thauera* (5.6%), albeit with no significant difference (WT; n = 12, *p* > 0.05). Further, even though the sequenced *nosZ* reads were less distributed to *Rhodothermus* (from 18.9% to 12.7%) in both two antibiotics-amended samples, the OTC group was observed to own comparatively diverse hosts such as *Caldilinea* and *Thauera* (~5%) and exclusively including 12.4% of *Fulvivirga* carrying *nosZ* (Fig. 3). This finding is consistent with other studies that suggest the stepwise processes, such as nitrification and anaerobic digestion, are largely determined by the composition of functional microbial groups more than resistance genes^36^,^37^,^38^. Some key functional gene hosts identified in this study, such as *Hypomicrobium, Caldilinea* and *Thauera*, were also reportedly reducing NO and N_2_O under antibiotics or toxicants stressed habitats^39^,^40^, where higher abundances of *Hypomicrobium* carrying *norB* and *nosZ* were reckoned to process a more complete denitrification^41^, probably because these two genes, compared to *nir, nap* and *nar* gene clusters, have comparatively low functional redundancy^42^ and therefore are likely to be the ceiling factors of the denitrifying process, especially under extreme conditions.

Our research suggests that the mechanisms underlying accumulation of nitrogen intermediates from denitrification in antibiotics-stressed conditions are majorly rooted in the composition of denitrifiers rather than the abundance of ARGs. It is still not very clear how OTC is less inhibitory than SMT to denitrifying bacteria. Nevertheless, the elevated abundances of some bacteria like *Hypomicrobium, Caldilinea* and *Thauera* potentially harbouring the *norB* and *nosZ* could be responsible for facilitating the dentrification process. The meta-sequencing results show that the enrichment of target resistance genes took place limitedly within *Pseudomonas* (Fig. 3). Recent studies also indicate that the dissemination of ARGs could not completely decouple from phylogeny and effectively transfer across habitats^43^. It is not uncommon that the resistome sequencing with ARGs integrated or propagated by plasmids could not effectively discern the relationship between these pseudo-genes to their originals^38^,^44^, future studies concerning the resistance detection of culturable bacteria therefore are highly suggested. Also, to more clearly reveal the mechanisms of the antibiotic-stressed denitrification, a more precisely operated experiment is needed particularly by calculating the N-balance with all nitrogen species included. At current stage, we could conclude that the facilitation of restoring a denitrification in landfilled refuses exposed to antibiotics on long term is more pronounced by the evolving bacterial community than by increasing ARG levels.

## Methods

### Sample collection and pretreatment

Laogang Municipal Waste Disposal Co., Ltd (Laogang Landfill) which is located in the outer southeast suburbs of Shanghai and typically receives approximately 20000 t/d MSWs across greater Shanghai, was selected as the sampling site. The landfilling procedure was operated by a multi-lift method, and each lift was ultimately covered with 0.5 m of sandy clay. The refuse was collected in triplicate using corers (Geoprobe 7822DT System, US). Every 25 g sample of core refuse was preincubated at 37 °C in a 250 ml rubber sealed serum bottle, and N_2_ was used to replace the headspace to keep an anaerobic condition. After one month of preincubation (to eliminate background NO_3_^−^-N and NO_2_^−^-N), the collected refuse samples were homogenized for the subsequent antibiotic-leachate incubation experiment. Landfill leachates were sampled from the leachate collection pipes of the subject landfill lifts, the composition of pretreated refuse and leachates is listed in Table 2. NH_3_-N in these leachate samples were precipitated by MPA methods as described before^45^, and replaced with the equivalent of NO_3_^−^, N (450 mg-N/L, Table 2).

### Measurement of nitrogen oxides (NO and N_2_O) and nitrogen (N_2_) gases

The refuse-antibiotic-leachate mixture (composition detailed in next section) was put in a 50 ml crimp neck borosilicate vial (CNW, Germany) sealed with a rubber septum and aluminum cap, and the vial was purged with helium (He) for 4 min using a timer-controlled multi-valve air pump (45 s in evacuation mode, 3 min 25 s in He filling mode). This procedure was repeated for 4 cycles prior to gas measurement according to the chamber incubation technique^46^. The headspace gas of each vial was collected by an autosampler every 3 hours, and the headspace samples were measured by gas chromatography (GC, Agilent 7890 A, USA) with two working columns: (i) 10 m poraPLOT U fitted with an electron capture detector (ECD) for N_2_O (detection limit, 0.1 ppb); and (ii) 20 m 5 Å Molsieve fitted with a thermal conductivity detector (TCD) for N_2_ (detection limit, 0.1 ppb). Column pressure (He) and temperature are 200 kPa and 36 °C, respectively, for the PLOT U column, and 250 kPa and 50 °C, respectively, for the Molsieve column. The NO concentration was measured by a chemiluminescence analyzer (Sievers Nitric Oxide Analyzer 280i, GE, USA) with a detection limit of 0.1 ppb. The emission fluxes of target gases over the tests were calculated using the following equation:


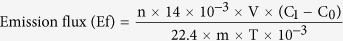


where the Ef denotes the target gas emission flux (μg-N/kg-refuse·h^−1^); n and V represent the number of nitrogen atoms (N) in each target gas molecule and headspace volume (V = 50 ml), respectively; and C_1_, C_0_, m and T are indicatives with respect to selected real-time concentration (ppm), initial concentration of target gas (ppm) in the headspace, dry refuse weight (g) and detection time, respectively. The real-time measurement data in the 24^th^ hour were selected for emission flux calculation when the concentration of target gases became stable (real-time target gases emission data of samples after 60-day antibiotics amendment were provided in the Fig. S5).

### Short-term antibiotic exposure experiments

This experiment was designed to mimic the acute effects of high dosage antibiotics on the denitrification in a landfill. The SMT and OTC (99.0%, Aladdin Industrial Inc., Shanghai) were dissolved in methanol (UPLC grade, CNW- GmbH, Germany) with a concentration of 250 mg/L and stored at −20 °C in a lab freezer. Before the incubation test, two bulk antibiotic solutions were diluted into a final concentration of 25 mg/L with MilliQ water. The combined solution (diluted antibiotic solution and pretreated leachate) were both purged with He for 10 min. The refuse-antibiotic-leachate mixture in each test vial consists of 5 g (dry weight) of homogenized raw (unexposed) refuse, 1 ml of pretreated leachate and 1 ml of antibiotic solution (15 mg/L, 1 ml of MilliQ water for control samples). The nitrogen oxides (NO and N_2_O) and N_2_ gases were measured consecutively for 24 hours as described above. The background levels of OTC and SMT in the tested inoculum were 0.24 ± 0.18 and 0.14 ± 0.07 mg/kg (n = 25), respectively (concentrations and method details given in the Supporting Information Table S6).

### Long-term antibiotic exposure experiment

This experiment was designed to test the hypothesis that long-term exposure (SMT and OTC) will alleviate the inhibition of antibiotics on the denitrification process (compared to the acute effects) in the landfill due to the development of AR. According to previous research^22^, additional antibiotics from new adding layer could transfer to the wastes located in previous sampling surfaces via landfill leachate. The piling-up of one waste-compacts layer above our sampling areas general costs two months, therefore the incubation period is designed as ~60 days. Our field study of lyophilized refuse samples (n = 25) sampled from two large landfills in the Great Shanghai Metropolis (Laogang landfill and Liming landfill) shows that the 25^th^ percentile, median, 75^th^ percentile, and maximum SMT and OTC concentrations are 0.13, 0.21, 0.27 and 0.7 mg/kg, respectively; and 0.12, 0.16, 0.21 and 0.27 mg/kg, respectively (Table S6). Thus, another 50 g of collected landfill refuse was initially amended with SMT and OTC at a loading rate equivalent to the median level (0.2 mg/kg for both groups) at 37 °C in a 250 ml rubber-sealed serum bottle. The antibiotics loading on the culture was subsequently increased to 0.5 mg/kg and 5.0 mg/kg on day 20 and day 45, respectively. During the antibiotics-exposure incubation, N_2_ was used to replace the headspace of the rubber-sealed serum bottle every day to maintain anaerobic conditions. The incubation procedure was ceased at Day 65 when the leachates were added in, and emission of nitrogen oxides from these long-term antibiotics-amended refuses was measured immediately.

### DNA Extraction and Sequencing Analysis

Samples collected in this study included raw landfill refuse (unexposed) and refuse-amended (long-term exposure) with SMT and OTC. Each sample was subdivided into two equivalent subsamples: one was used to calculate the physical composition of the refuse samples, another was centrifuged at 5000 rpm for 10 min (removing extra liquid), and 200 mg of the pellet was collected for DNA extraction using a PowerSoil DNA Isolation Kit (MOBIO, USA) according to the manufacturer’s protocol. The yield and quality of the DNA extractions were verified by spectrophotometry (Nanodrop 1000), requiring that extracts with the OD_260/280_ value between 1.8 and 2.0 be used to ensure that no sequencing inhibitors were present. The qualified DNA extracts were stored at −20 °C for further analysis.

The resulting DNA extracts were provided in duplicate for library construction and high-throughput sequencing with the Illumina HiSeq 2000 platform. Approximately 5 Gb (giga base pairs) of data were generated for each sample. The raw metagenomic data were initially trimmed using Trimmomatic to remove reads with adapter contamination, with three or more ambiguous nucleotides, or with an average quality score below 15 but over 36 bp, and the reads were further filtered and trimmed by using Stickle (Version 1.33) to remove reads that did not meet the Q_20_ requirement (>50% reads; https://github.com/najoshi/sickle). The quality of the filtered data and the number of clean reads are both described in detail in the Supporting Information (Table S7). The remaining clean reads were used for further analysis: the putative ARGs were searched against an antibiotic resistance database (ARDB) with an *E* value of ≤10^−5^. For exploring denitrifying genes in refuse, the quality-filtered Illumina reads of different samples were separately aligned to local nucleotide databases created by downloading DNA sequences of *napG(HJ*), *narA(B*), *nirK(S), norB* and *nosZ* genes from RDP FunGene (http://fungene.cme.msu.edu/). The identification of the bacterial hosts of the ARGs and denitrifying genes was subjected to BLASTx against the NCBI-nr database with E-value cut-off at 10^−5^. The assigned sequences were then visualized by MEGAN with the Lowest Common Ancestor (using default parameters) algorithm as described in previous research^47^. The quality-filtered Illumina reads of all six refuse samples were submitted to the European Bioinformatics Institute server (accession number: ERS1069313).

### Statistics

This study contrasted unexposed (raw refuses), SMT-exposed and OTC-exposed samples relative to the variation of target gases, functional genes, and microbial community using two independent metagenomes per incubation condition (6 samples in total). Prior to conducting statistical comparisons among the three groups, data duplicates of each group were standardized and tested to determine whether they were statistically the same by t-test^23^. If the replica were statistically the same, duplicates were clumped as averages to perform more rigorous statistical comparisons including two-sample testing (paired t-test) between unexposed and exposed samples, Wilcoxon tests for non-normally distributed subset data, and analysis of variation (ANOVA) among three incubation samples and/or different target gases/genes. Data analysis and statistical assessments were performed using SPSS 22 software (SPSS, Inc., Chicago, IL), and statistical significance was defined by 95% confidence intervals (p < 0.05, two-tail). Principal component analysis (PCA) indicating difference among samples was employed in PAST (http://folk.uio.no/ohammer/past/). The Mantel test and Variance Partitioning Analysis (VPA) were applied to evaluate the correlations among microbial communities with environmental variables and to evaluate the contributions of functional genes and denitrifying bacterial diversity to target gas emission patterns by using RStudio, respectively. Circos graphs were produced via Circos software on line (http://circos.ca/).

## Additional Information

**How to cite this article**: Wu, D. *et al*. Change in microbial community in landfill refuse contaminated with antibiotics facilitates denitrification more than the increase in ARG over long-term. *Sci. Rep.* **7**, 41230; doi: 10.1038/srep41230 (2017).

**Publisher's note:** Springer Nature remains neutral with regard to jurisdictional claims in published maps and institutional affiliations.

## Supplementary Material

Supplementary Information

## Figures and Tables

**Table 1:** Relative abundance of denitrification functional genes in different samples.

Functional genes	Abundance (genes/total of each sample; Mean ± SD, n = 2)		
	Control	Long-term SMT exposed	Long-term OTC exposed
*narG*	4.08E-04 ± 1.77E-05	2.23E-04 ± 2.40E-05	3.12E-04 ± 1.84E-05
*narH*	1.74E-04 ± 8.49E-06	1.23E-04 ± 6.36E-06	1.33E-04 ± 7.07E-07
*narI*	7.55E-05 ± 8.49E-06	6.05E-05 ± 1.53E-05	9.14E-05 ± 1.36E-05
*narJ*	0.00E + 00 ± 0.00E + 00	1.02E-06 ± 1.44E-06	0.00E + 00 ± 0.00E + 00
*napA*	3.16E-05 ± 7.07E-07	4.48E-05 ± 5.80E-06	3.49E-05 ± 3.39E-06
Nitrate + Reduced acceptor <=> Nitrite + Acceptor + H_2_O			
*nirK*	2.38E-04 ± 4.81E-05	1.57E-04 ± 2.83E-06	1.99E-04 ± 1.48E-05
*nirS*	7.42E-05 ± 1.41E-07	6.55E-05 ± 3.32E-06	8.62E-05 ± 3.11E-06
Nitrite + Reduced azurin + H+ <=> Nitric oxide + H_2_O + Oxidized azurin			
*norB*	1.86E-04 ± 5.66E-06	1.51E-04 ± 2.33E-05	2.24E-04 ± 7.07E-06
2 Nitric oxide + 2 Ferrocytochrome c + 2H+ <=> Nitrous oxide + 2 Ferricytochrome c + H_2_O			
*nosZ*	2.01E-04 ± 1.41E-06	1.08E-04 ± 6.36E-06	1.78E-04 ± 1.34E-05
Nitrous oxide + 2 Ferrocytochrome c + 2H+ <=> Nitrogen + 2 Ferricytochrome c + H_2_O			

**Table 2:** Characteristics of landfill refuse and leachate (Mean ± SD, n = 4).

Refuse	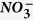 -N	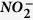 -N	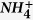 -N	Sand	Slit	Clay	Moisture
(Units)	mg/kg-refuse			(%)			
Raw-unexposed	0.12 (0.03)	n.a	3.2 (1.0)	16.4 (5.4)	9.3 (4.7)	75.0 (7.2)	23.7 (4.3)
SMT-exposed	n.a.	n.a.	3.3 (0.2)	24.5 (10.4)	19.1 (8.6)	56.3 (14.7)	15.8 (6.3)
OTC-exposed	0.03	n.a.	3.8 (0.3)	15.1 (5.8)	10.1 (3.9)	76.9 (4.2)	22.4 (5.5)
Leachate*	16.2 (10.2)	1.2 (0.2)	625.6 (37.2)	n.a			
Leachate-t	451.2 (13.6)	3.7 (0.4)	22.69 (9.2)				

^*^The unit of leachate is in mg-N/L; the pH values of raw and treated leachate are 7.8 and 8.5 respectively.
