# Supplementary material for: Change in microbial community in landfill refuse contaminated with antibiotics facilitates denitrification more than the increase in ARG over long-term
Source: Sci Rep. 2017 Jan 25;7:41230. doi: 10.1038/srep41230 (PMC5264584; doi:10.1038/srep41230)
Supplement: Supplementary Information [file srep41230-s1.doc]

**Change in microbial community in landfill refuse contaminated with antibiotics facilitates denitrification more than the increase in ARG over long term**

**Dong Wu^1,2^, Guanzhou Chen^3^, Xiaojun Zhang^3^, Kai Yang^1,2^, Bing Xie^1,2*^**

**^1^** Key Laboratory for Urban Ecological Processes and Eco-Restoration, School of Ecological and Environmental Science, East China Normal University, Shanghai 200241, China

**^2^** Joint Research Institute for New Energy and the Environment, East China Normal University and Colorado State University, Shanghai 200062, China

**^3^** State Key Laboratory of Microbial Metabolism, School of Life Sciences and Biotechnology, Shanghai Jiao Tong University, Shanghai 200240, China

*Corresponding Author: Bing Xie

East China Normal University

Shanghai 200062

P.R. China

Email: [bxie@des.ecnu.edu.cn](mailto:bxie@des.ecnu.edu.cn)

Phone: (+86) 021-54341276

**SI-1 Sequencing results and additional statistical analysis**

The raw sequence number and abundance of ARGs and genes of interest were provided in this section.

Diagrams indicating variation of microbial and functional genes and to what extend the gases emission pattern can be explained were all provided in this section.

## Table S1 Relative abundance of each ARG type in the respective raw samples

| **ARG Type** | **ARG Sub-type** | **A1** | **A2** | **B1** | **B2** | **C1** | **C2** | **A1%** | **A2%** | **B1%** | **B2%** | **C1%** | **C2%** |
| --- | --- | --- | --- | --- | --- | --- | --- | --- | --- | --- | --- | --- | --- |
| **aminoglycoside** | aac3x | 0 | 0 | 0 | 0 | 0 | 2 | 0.00 | 0.00 | 0.00 | 0.00 | 0.00 | 0.06 |
|  | aac3iv | 10 | 6 | 3 | 6 | 3 | 5 | 0.31 | 0.17 | 0.07 | 0.13 | 0.09 | 0.15 |
|  | aac2i | 0 | 0 | 0 | 0 | 0 | 1 | 0.00 | 0.00 | 0.00 | 0.00 | 0.00 | 0.03 |
|  | aac3iia | 5 | 2 | 3 | 1 | 8 | 2 | 0.15 | 0.06 | 0.07 | 0.02 | 0.25 | 0.06 |
|  | ant2ib | 0 | 0 | 1 | 0 | 0 | 1 | 0.00 | 0.00 | 0.02 | 0.00 | 0.00 | 0.03 |
|  | aac2ic | 0 | 0 | 0 | 1 | 0 | 0 | 0.00 | 0.00 | 0.00 | 0.02 | 0.00 | 0.00 |
|  | aac2ib | 1 | 1 | 1 | 0 | 0 | 0 | 0.03 | 0.03 | 0.02 | 0.00 | 0.00 | 0.00 |
|  | ant2ia | 5 | 19 | 59 | 50 | 4 | 10 | 0.15 | 0.55 | 1.43 | 1.11 | 0.13 | 0.30 |
|  | aph3iiia | 0 | 2 | 2 | 0 | 0 | 0 | 0.00 | 0.06 | 0.05 | 0.00 | 0.00 | 0.00 |
|  | aac3iii | 0 | 0 | 2 | 0 | 0 | 1 | 0.00 | 0.00 | 0.05 | 0.00 | 0.00 | 0.03 |
|  | aph3ib | 4 | 3 | 4 | 2 | 2 | 1 | 0.12 | 0.09 | 0.10 | 0.04 | 0.06 | 0.03 |
|  | aac3ix | 0 | 0 | 0 | 0 | 0 | 1 | 0.00 | 0.00 | 0.00 | 0.00 | 0.00 | 0.03 |
|  | aac6i | 0 | 0 | 0 | 1 | 0 | 0 | 0.00 | 0.00 | 0.00 | 0.02 | 0.00 | 0.00 |
|  | aac3vii | 0 | 1 | 1 | 0 | 0 | 1 | 0.00 | 0.03 | 0.02 | 0.00 | 0.00 | 0.03 |
|  | aph3ia | 0 | 0 | 0 | 1 | 2 | 1 | 0.00 | 0.00 | 0.00 | 0.02 | 0.06 | 0.03 |
|  | aac6ib | 13 | 13 | 39 | 50 | 5 | 8 | 0.40 | 0.37 | 0.94 | 1.11 | 0.16 | 0.24 |
|  | aac6iia | 1 | 0 | 1 | 1 | 2 | 0 | 0.03 | 0.00 | 0.02 | 0.02 | 0.06 | 0.00 |
|  | aac3ia | 18 | 26 | 28 | 40 | 4 | 8 | 0.55 | 0.75 | 0.68 | 0.89 | 0.13 | 0.24 |
|  | aac6ie | 0 | 4 | 0 | 0 | 1 | 0 | 0.00 | 0.12 | 0.00 | 0.00 | 0.03 | 0.00 |
|  | aac3iib | 0 | 0 | 0 | 0 | 1 | 0 | 0.00 | 0.00 | 0.00 | 0.00 | 0.03 | 0.00 |
|  | ant3ia | 26 | 34 | 142 | 114 | 18 | 14 | 0.79 | 0.98 | 3.44 | 2.54 | 0.57 | 0.42 |
|  | aad9ib | 0 | 0 | 0 | 2 | 0 | 1 | 0.00 | 0.00 | 0.00 | 0.04 | 0.00 | 0.03 |
|  | aph33ib | 2 | 3 | 16 | 20 | 1 | 0 | 0.06 | 0.09 | 0.39 | 0.44 | 0.03 | 0.00 |
|  | aph6ic | 6 | 13 | 3 | 6 | 0 | 1 | 0.18 | 0.37 | 0.07 | 0.13 | 0.00 | 0.03 |
|  | aph6id | 4 | 8 | 37 | 31 | 2 | 4 | 0.12 | 0.23 | 0.90 | 0.69 | 0.06 | 0.12 |
|  | aph6ib | 0 | 0 | 0 | 1 | 0 | 0 | 0.00 | 0.00 | 0.00 | 0.02 | 0.00 | 0.00 |
|  | str | 0 | 0 | 0 | 0 | 2 | 0 | 0.00 | 0.00 | 0.00 | 0.00 | 0.06 | 0.00 |
|  | aph6ia | 0 | 0 | 0 | 0 | 0 | 1 | 0.00 | 0.00 | 0.00 | 0.00 | 0.00 | 0.03 |
| **bacitracin** | baca | 2604 | 2684 | 3095 | 3195 | 2048 | 2193 | 79.60 | 77.36 | 74.92 | 71.08 | 64.67 | 65.66 |
|  | bcra | 2 | 11 | 15 | 19 | 10 | 10 | 0.06 | 0.32 | 0.36 | 0.42 | 0.32 | 0.30 |
| **beta-lactam** | bl3_shw | 2 | 2 | 3 | 1 | 7 | 4 | 0.06 | 0.06 | 0.07 | 0.02 | 0.22 | 0.12 |
|  | bl1_fox | 0 | 0 | 1 | 0 | 0 | 0 | 0.00 | 0.00 | 0.02 | 0.00 | 0.00 | 0.00 |
|  | bl1_mox | 0 | 0 | 1 | 0 | 0 | 0 | 0.00 | 0.00 | 0.02 | 0.00 | 0.00 | 0.00 |
|  | bl1_sm | 0 | 0 | 1 | 1 | 0 | 0 | 0.00 | 0.00 | 0.02 | 0.02 | 0.00 | 0.00 |
|  | bl2e_y56 | 0 | 0 | 0 | 1 | 0 | 1 | 0.00 | 0.00 | 0.00 | 0.02 | 0.00 | 0.03 |
|  | bl2b_tem | 0 | 0 | 0 | 5 | 1 | 0 | 0.00 | 0.00 | 0.00 | 0.11 | 0.03 | 0.00 |
|  | bl2b_tem2 | 4 | 0 | 0 | 0 | 0 | 0 | 0.12 | 0.00 | 0.00 | 0.00 | 0.00 | 0.00 |
|  | bl3_vim | 0 | 0 | 2 | 2 | 2 | 2 | 0.00 | 0.00 | 0.05 | 0.04 | 0.06 | 0.06 |
|  | bl3_l | 1 | 1 | 4 | 2 | 0 | 0 | 0.03 | 0.03 | 0.10 | 0.04 | 0.00 | 0.00 |
|  | bl2d_oxa10 | 0 | 0 | 6 | 2 | 0 | 0 | 0.00 | 0.00 | 0.15 | 0.04 | 0.00 | 0.00 |
|  | bl2be_per | 3 | 3 | 1 | 0 | 0 | 0 | 0.09 | 0.09 | 0.02 | 0.00 | 0.00 | 0.00 |
|  | bl1_acc | 0 | 0 | 0 | 0 | 0 | 1 | 0.00 | 0.00 | 0.00 | 0.00 | 0.00 | 0.03 |
|  | bl2be_ctxm | 0 | 0 | 1 | 3 | 0 | 0 | 0.00 | 0.00 | 0.02 | 0.07 | 0.00 | 0.00 |
|  | pbp2 | 2 | 2 | 0 | 2 | 1 | 0 | 0.06 | 0.06 | 0.00 | 0.04 | 0.03 | 0.00 |
|  | pbp2b | 3 | 0 | 0 | 4 | 1 | 0 | 0.09 | 0.00 | 0.00 | 0.09 | 0.03 | 0.00 |
|  | pbp1a | 0 | 3 | 1 | 0 | 0 | 0 | 0.00 | 0.09 | 0.02 | 0.00 | 0.00 | 0.00 |
|  | bl2a_iii2 | 0 | 0 | 0 | 1 | 0 | 0 | 0.00 | 0.00 | 0.00 | 0.02 | 0.00 | 0.00 |
|  | bl2_kpc | 0 | 0 | 0 | 0 | 1 | 0 | 0.00 | 0.00 | 0.00 | 0.00 | 0.03 | 0.00 |
|  | bl2b_tle | 0 | 0 | 0 | 1 | 1 | 0 | 0.00 | 0.00 | 0.00 | 0.02 | 0.03 | 0.00 |
|  | bl2b_ula | 0 | 0 | 0 | 1 | 0 | 0 | 0.00 | 0.00 | 0.00 | 0.02 | 0.00 | 0.00 |
|  | bl2d_oxa1 | 2 | 0 | 0 | 0 | 0 | 0 | 0.06 | 0.00 | 0.00 | 0.00 | 0.00 | 0.00 |
|  | bl2d_oxa2 | 5 | 3 | 11 | 12 | 3 | 11 | 0.15 | 0.09 | 0.27 | 0.27 | 0.09 | 0.33 |
|  | bl2d_lcr1 | 0 | 0 | 0 | 1 | 0 | 0 | 0.00 | 0.00 | 0.00 | 0.02 | 0.00 | 0.00 |
|  | bl2d_moxa | 0 | 0 | 0 | 0 | 1 | 0 | 0.00 | 0.00 | 0.00 | 0.00 | 0.03 | 0.00 |
|  | bl2d_r39 | 0 | 0 | 0 | 0 | 0 | 3 | 0.00 | 0.00 | 0.00 | 0.00 | 0.00 | 0.09 |
| **chloramphenicol** | cata1 | 8 | 0 | 4 | 0 | 0 | 0 | 0.24 | 0.00 | 0.10 | 0.00 | 0.00 | 0.00 |
|  | ceoa | 3 | 0 | 1 | 0 | 4 | 1 | 0.09 | 0.00 | 0.02 | 0.00 | 0.13 | 0.03 |
|  | ceob | 125 | 122 | 202 | 220 | 117 | 124 | 3.82 | 3.52 | 4.89 | 4.89 | 3.69 | 3.71 |
|  | cml_e3 | 8 | 2 | 27 | 29 | 14 | 13 | 0.24 | 0.06 | 0.65 | 0.65 | 0.44 | 0.39 |
|  | cml_e8 | 3 | 6 | 5 | 5 | 4 | 0 | 0.09 | 0.17 | 0.12 | 0.11 | 0.13 | 0.00 |
|  | catb3 | 0 | 0 | 7 | 12 | 4 | 3 | 0.00 | 0.00 | 0.17 | 0.27 | 0.13 | 0.09 |
|  | cml_e1 | 0 | 0 | 11 | 10 | 5 | 4 | 0.00 | 0.00 | 0.27 | 0.22 | 0.16 | 0.12 |
|  | cml_e2 | 0 | 0 | 1 | 1 | 0 | 0 | 0.00 | 0.00 | 0.02 | 0.02 | 0.00 | 0.00 |
|  | cata16 | 0 | 0 | 0 | 4 | 0 | 2 | 0.00 | 0.00 | 0.00 | 0.09 | 0.00 | 0.06 |
|  | cml_e7 | 0 | 0 | 0 | 0 | 1 | 0 | 0.00 | 0.00 | 0.00 | 0.00 | 0.03 | 0.00 |
|  | cml_e4 | 0 | 0 | 0 | 0 | 0 | 2 | 0.00 | 0.00 | 0.00 | 0.00 | 0.00 | 0.06 |
| **fluoroquinolone** | mfpa | 5 | 1 | 0 | 5 | 0 | 0 | 0.15 | 0.03 | 0.00 | 0.11 | 0.00 | 0.00 |
|  | smed | 24 | 15 | 20 | 22 | 13 | 19 | 0.73 | 0.43 | 0.48 | 0.49 | 0.41 | 0.57 |
|  | smee | 253 | 223 | 254 | 227 | 159 | 187 | 7.73 | 6.43 | 6.15 | 5.05 | 5.02 | 5.60 |
|  | smef | 3 | 1 | 4 | 1 | 0 | 2 | 0.09 | 0.03 | 0.10 | 0.02 | 0.00 | 0.06 |
|  | smeb | 0 | 2 | 4 | 7 | 1 | 2 | 0.00 | 0.06 | 0.10 | 0.16 | 0.03 | 0.06 |
|  | smec | 0 | 0 | 0 | 0 | 0 | 1 | 0.00 | 0.00 | 0.00 | 0.00 | 0.00 | 0.03 |
| **MLS** | erea | 7 | 10 | 33 | 50 | 4 | 10 | 0.21 | 0.29 | 0.80 | 1.11 | 0.13 | 0.30 |
|  | ermb | 2 | 1 | 6 | 7 | 8 | 0 | 0.06 | 0.03 | 0.15 | 0.16 | 0.25 | 0.00 |
|  | macb | 142 | 160 | 183 | 225 | 160 | 158 | 4.34 | 4.61 | 4.43 | 5.01 | 5.05 | 4.73 |
|  | mefa | 20 | 13 | 65 | 46 | 23 | 18 | 0.61 | 0.37 | 1.57 | 1.02 | 0.73 | 0.54 |
|  | mpha | 0 | 0 | 4 | 1 | 1 | 3 | 0.00 | 0.00 | 0.10 | 0.02 | 0.03 | 0.09 |
|  | ermc | 1 | 3 | 2 | 2 | 0 | 0 | 0.03 | 0.09 | 0.05 | 0.04 | 0.00 | 0.00 |
|  | ermx | 2 | 1 | 2 | 7 | 2 | 2 | 0.06 | 0.03 | 0.05 | 0.16 | 0.06 | 0.06 |
|  | oleb | 1 | 0 | 0 | 0 | 0 | 1 | 0.03 | 0.00 | 0.00 | 0.00 | 0.00 | 0.03 |
|  | norm | 0 | 0 | 2 | 3 | 0 | 1 | 0.00 | 0.00 | 0.05 | 0.07 | 0.00 | 0.03 |
|  | cara | 1 | 4 | 0 | 0 | 0 | 0 | 0.03 | 0.12 | 0.00 | 0.00 | 0.00 | 0.00 |
|  | ermg | 23 | 16 | 22 | 33 | 13 | 10 | 0.70 | 0.46 | 0.53 | 0.73 | 0.41 | 0.30 |
|  | erma | 4 | 11 | 6 | 11 | 4 | 5 | 0.12 | 0.32 | 0.15 | 0.24 | 0.13 | 0.15 |
|  | ermf | 36 | 17 | 141 | 220 | 88 | 117 | 1.10 | 0.49 | 3.41 | 4.89 | 2.78 | 3.50 |
|  | ermv | 0 | 1 | 0 | 0 | 0 | 0 | 0.00 | 0.03 | 0.00 | 0.00 | 0.00 | 0.00 |
|  | ermy | 0 | 0 | 1 | 5 | 0 | 0 | 0.00 | 0.00 | 0.02 | 0.11 | 0.00 | 0.00 |
|  | srmb | 0 | 0 | 4 | 0 | 0 | 1 | 0.00 | 0.00 | 0.10 | 0.00 | 0.00 | 0.03 |
| **multi-durg** | acrb | 99 | 75 | 143 | 200 | 111 | 99 | 3.03 | 2.16 | 3.46 | 4.45 | 3.51 | 2.96 |
|  | amrb | 21 | 18 | 57 | 51 | 17 | 17 | 0.64 | 0.52 | 1.38 | 1.13 | 0.54 | 0.51 |
|  | opra | 2 | 0 | 1 | 1 | 2 | 1 | 0.06 | 0.00 | 0.02 | 0.02 | 0.06 | 0.03 |
|  | mexy | 5 | 8 | 28 | 36 | 14 | 13 | 0.15 | 0.23 | 0.68 | 0.80 | 0.44 | 0.39 |
|  | mexx | 0 | 1 | 2 | 3 | 0 | 0 | 0.00 | 0.03 | 0.05 | 0.07 | 0.00 | 0.00 |
|  | mexa | 2 | 3 | 21 | 27 | 4 | 5 | 0.06 | 0.09 | 0.51 | 0.60 | 0.13 | 0.15 |
|  | oprm | 0 | 1 | 19 | 17 | 3 | 3 | 0.00 | 0.03 | 0.46 | 0.38 | 0.09 | 0.09 |
|  | adeb | 7 | 9 | 11 | 11 | 11 | 7 | 0.21 | 0.26 | 0.27 | 0.24 | 0.35 | 0.21 |
|  | mexf | 159 | 199 | 404 | 471 | 204 | 191 | 4.86 | 5.74 | 9.78 | 10.48 | 6.44 | 5.72 |
|  | oprn | 1 | 1 | 17 | 17 | 1 | 5 | 0.03 | 0.03 | 0.41 | 0.38 | 0.03 | 0.15 |
|  | mdtf | 8 | 12 | 16 | 13 | 4 | 5 | 0.24 | 0.35 | 0.39 | 0.29 | 0.13 | 0.15 |
|  | mexe | 1 | 4 | 19 | 21 | 4 | 11 | 0.03 | 0.12 | 0.46 | 0.47 | 0.13 | 0.33 |
|  | mexd | 26 | 53 | 203 | 232 | 44 | 52 | 0.79 | 1.53 | 4.91 | 5.16 | 1.39 | 1.56 |
|  | oprj | 3 | 1 | 25 | 54 | 7 | 5 | 0.09 | 0.03 | 0.61 | 1.20 | 0.22 | 0.15 |
|  | tolc | 0 | 2 | 0 | 0 | 1 | 0 | 0.00 | 0.06 | 0.00 | 0.00 | 0.03 | 0.00 |
|  | acra | 7 | 4 | 5 | 4 | 2 | 2 | 0.21 | 0.12 | 0.12 | 0.09 | 0.06 | 0.06 |
|  | mdr | 1 | 2 | 5 | 1 | 1 | 0 | 0.03 | 0.06 | 0.12 | 0.02 | 0.03 | 0.00 |
|  | mexi | 37 | 28 | 33 | 51 | 28 | 23 | 1.13 | 0.81 | 0.80 | 1.13 | 0.88 | 0.69 |
|  | mexw | 42 | 65 | 125 | 119 | 60 | 53 | 1.28 | 1.87 | 3.03 | 2.65 | 1.89 | 1.59 |
|  | mdtk | 0 | 0 | 0 | 7 | 3 | 0 | 0.00 | 0.00 | 0.00 | 0.16 | 0.09 | 0.00 |
|  | mexc | 0 | 0 | 0 | 1 | 0 | 0 | 0.00 | 0.00 | 0.00 | 0.02 | 0.00 | 0.00 |
|  | mexb | 43 | 57 | 166 | 174 | 71 | 78 | 1.31 | 1.64 | 4.02 | 3.87 | 2.24 | 2.34 |
| **sulfonamide** | sul1 | 98 | 111 | 704 | 830 | 99 | 112 | 3.00 | 3.20 | 17.04 | 18.47 | 3.13 | 3.35 |
|  | sul2 | 272 | 265 | 1195 | 1312 | 192 | 181 | 8.31 | 7.64 | 28.93 | 29.19 | 6.06 | 5.42 |
|  | sul3 | 1 | 0 | 8 | 10 | 0 | 1 | 0.03 | 0.00 | 0.19 | 0.22 | 0.00 | 0.03 |
| **tetracycline** | otra | 7 | 8 | 7 | 7 | 10 | 4 | 0.21 | 0.23 | 0.17 | 0.16 | 0.32 | 0.12 |
|  | tet | 9 | 11 | 9 | 13 | 6 | 6 | 0.28 | 0.32 | 0.22 | 0.29 | 0.19 | 0.18 |
|  | tet36 | 6 | 6 | 356 | 319 | 56 | 54 | 0.18 | 0.17 | 8.62 | 7.10 | 1.77 | 1.62 |
|  | tetc | 11 | 2 | 214 | 195 | 0 | 0 | 0.34 | 0.06 | 5.18 | 4.34 | 0.00 | 0.00 |
|  | tetg | 7 | 1 | 25 | 24 | 8 | 11 | 0.21 | 0.03 | 0.61 | 0.53 | 0.25 | 0.33 |
|  | tetm | 2 | 4 | 4 | 6 | 2 | 0 | 0.06 | 0.12 | 0.10 | 0.13 | 0.06 | 0.00 |
|  | teto | 1 | 1 | 0 | 3 | 1 | 0 | 0.03 | 0.03 | 0.00 | 0.07 | 0.03 | 0.00 |
|  | tetpa | 1 | 0 | 2 | 3 | 1 | 2 | 0.03 | 0.00 | 0.05 | 0.07 | 0.03 | 0.06 |
|  | tetpb | 19 | 34 | 35 | 37 | 23 | 22 | 0.58 | 0.98 | 0.85 | 0.82 | 0.73 | 0.66 |
|  | tett | 4 | 8 | 7 | 5 | 0 | 0 | 0.12 | 0.23 | 0.17 | 0.11 | 0.00 | 0.00 |
|  | tetv | 1 | 5 | 3 | 4 | 2 | 3 | 0.03 | 0.14 | 0.07 | 0.09 | 0.06 | 0.09 |
|  | tetw | 1 | 2 | 0 | 6 | 1 | 0 | 0.03 | 0.06 | 0.00 | 0.13 | 0.03 | 0.00 |
|  | tet30 | 0 | 1 | 0 | 0 | 0 | 0 | 0.00 | 0.03 | 0.00 | 0.00 | 0.00 | 0.00 |
|  | tet40 | 0 | 1 | 1 | 1 | 0 | 1 | 0.00 | 0.03 | 0.02 | 0.02 | 0.00 | 0.03 |
|  | tets | 0 | 1 | 0 | 1 | 0 | 1 | 0.00 | 0.03 | 0.00 | 0.02 | 0.00 | 0.03 |
|  | tetx | 0 | 10 | 42 | 47 | 5 | 3 | 0.00 | 0.29 | 1.02 | 1.05 | 0.16 | 0.09 |
|  | tet33 | 0 | 0 | 3 | 7 | 0 | 0 | 0.00 | 0.00 | 0.07 | 0.16 | 0.00 | 0.00 |
|  | tet37 | 0 | 0 | 5 | 8 | 0 | 3 | 0.00 | 0.00 | 0.12 | 0.18 | 0.00 | 0.09 |
|  | tet39 | 0 | 0 | 1 | 0 | 0 | 0 | 0.00 | 0.00 | 0.02 | 0.00 | 0.00 | 0.00 |
|  | teta | 0 | 0 | 4 | 0 | 0 | 0 | 0.00 | 0.00 | 0.10 | 0.00 | 0.00 | 0.00 |
|  | tetq | 0 | 0 | 6 | 3 | 2 | 6 | 0.00 | 0.00 | 0.15 | 0.07 | 0.06 | 0.18 |
|  | tet34 | 0 | 0 | 0 | 1 | 0 | 0 | 0.00 | 0.00 | 0.00 | 0.02 | 0.00 | 0.00 |
|  | tet41 | 0 | 0 | 0 | 0 | 1 | 1 | 0.00 | 0.00 | 0.00 | 0.00 | 0.03 | 0.03 |
|  | tetl | 0 | 0 | 0 | 0 | 1 | 0 | 0.00 | 0.00 | 0.00 | 0.00 | 0.03 | 0.00 |
| **Vancomycin** | vana | 5 | 9 | 10 | 11 | 7 | 10 | 0.15 | 0.26 | 0.24 | 0.24 | 0.22 | 0.30 |
|  | vand | 0 | 5 | 0 | 3 | 2 | 0 | 0.00 | 0.14 | 0.00 | 0.07 | 0.06 | 0.00 |
|  | vanrd | 0 | 1 | 0 | 1 | 0 | 1 | 0.00 | 0.03 | 0.00 | 0.02 | 0.00 | 0.03 |
|  | vanxd | 0 | 0 | 2 | 0 | 1 | 1 | 0.00 | 0.00 | 0.05 | 0.00 | 0.03 | 0.03 |
|  | vansd | 0 | 0 | 0 | 1 | 0 | 2 | 0.00 | 0.00 | 0.00 | 0.02 | 0.00 | 0.06 |
|  | vanya | 0 | 0 | 0 | 0 | 0 | 1 | 0.00 | 0.00 | 0.00 | 0.00 | 0.00 | 0.03 |
|  | vanc | 4 | 2 | 2 | 2 | 3 | 3 | 0.12 | 0.06 | 0.05 | 0.04 | 0.09 | 0.09 |
|  | vang | 2 | 0 | 2 | 4 | 5 | 2 | 0.06 | 0.00 | 0.05 | 0.09 | 0.16 | 0.06 |
|  | vanrg | 4 | 1 | 4 | 10 | 2 | 7 | 0.12 | 0.03 | 0.10 | 0.22 | 0.06 | 0.21 |
|  | vansc | 1 | 1 | 0 | 1 | 0 | 2 | 0.03 | 0.03 | 0.00 | 0.02 | 0.00 | 0.06 |
|  | vanwb | 1 | 2 | 3 | 0 | 2 | 1 | 0.03 | 0.06 | 0.07 | 0.00 | 0.06 | 0.03 |
|  | vanxb | 1 | 0 | 0 | 0 | 0 | 0 | 0.03 | 0.00 | 0.00 | 0.00 | 0.00 | 0.00 |
|  | vanrc | 0 | 1 | 3 | 2 | 3 | 4 | 0.00 | 0.03 | 0.07 | 0.04 | 0.09 | 0.12 |
|  | vansg | 0 | 1 | 1 | 1 | 1 | 0 | 0.00 | 0.03 | 0.02 | 0.02 | 0.03 | 0.00 |
|  | vantg | 0 | 1 | 0 | 0 | 0 | 0 | 0.00 | 0.03 | 0.00 | 0.00 | 0.00 | 0.00 |
|  | vanxyc | 0 | 0 | 0 | 1 | 0 | 0 | 0.00 | 0.00 | 0.00 | 0.02 | 0.00 | 0.00 |
|  | vanyg | 0 | 0 | 0 | 1 | 0 | 0 | 0.00 | 0.00 | 0.00 | 0.02 | 0.00 | 0.00 |
|  | vanb | 0 | 0 | 0 | 0 | 2 | 1 | 0.00 | 0.00 | 0.00 | 0.00 | 0.06 | 0.03 |
|  | vanrb | 0 | 0 | 0 | 0 | 1 | 0 | 0.00 | 0.00 | 0.00 | 0.00 | 0.03 | 0.00 |
|  | vanxyg | 0 | 0 | 0 | 0 | 1 | 0 | 0.00 | 0.00 | 0.00 | 0.00 | 0.03 | 0.00 |
|  | vanha | 2 | 3 | 4 | 2 | 3 | 4 | 0.06 | 0.09 | 0.10 | 0.04 | 0.09 | 0.12 |
|  | vanra | 5 | 8 | 11 | 8 | 11 | 13 | 0.15 | 0.23 | 0.27 | 0.18 | 0.35 | 0.39 |
|  | vansa | 4 | 3 | 16 | 7 | 12 | 20 | 0.12 | 0.09 | 0.39 | 0.16 | 0.38 | 0.60 |
|  | vanxa | 1 | 0 | 3 | 2 | 0 | 2 | 0.03 | 0.00 | 0.07 | 0.04 | 0.00 | 0.06 |

## Table S2 Relative abundance of each denitrification functional genes in the respective raw samples

|  | Reads (genes) | | | | | | Abundance (genes/total of each sample) | | | | | |
| --- | --- | --- | --- | --- | --- | --- | --- | --- | --- | --- | --- | --- |
|  | A1 | A2 | B1 | B2 | C1 | C2 | A1 | A2 | B1 | B2 | C1 | C2 |
| narG | 165 | 170 | 190 | 168 | 91 | 118 | 3.95E-04 | 4.20E-04 | 3.25E-04 | 2.99E-04 | 2.06E-04 | 2.40E-04 |
| narH | 70 | 73 | 78 | 74 | 56 | 58 | 1.68E-04 | 1.80E-04 | 1.33E-04 | 1.32E-04 | 1.27E-04 | 1.18E-04 |
| narI | 29 | 33 | 59 | 46 | 22 | 35 | 6.95E-05 | 8.15E-05 | 1.01E-04 | 8.18E-05 | 4.97E-05 | 7.13E-05 |
| narJ | 0 | 0 | 0 | 0 | 0 | 1 | 0.00E+00 | 0.00E+00 | 0.00E+00 | 0.00E+00 | 0.00E+00 | 2.04E-06 |
| napA | 13 | 13 | 19 | 21 | 18 | 24 | 3.11E-05 | 3.21E-05 | 3.25E-05 | 3.73E-05 | 4.07E-05 | 4.89E-05 |
| napB | 0 | 0 | 0 | 0 | 0 | 0 | 0.00E+00 | 0.00E+00 | 0.00E+00 | 0.00E+00 | 0.00E+00 | 0.00E+00 |
| Nitrate + Reduced acceptor <=> Nitrite + Acceptor + H2O; | | | | | | | | | | | | |
| nirK | 85 | 110 | 122 | 106 | 82 | 93 | 2.04E-04 | 2.72E-04 | 2.09E-04 | 1.88E-04 | 1.85E-04 | 1.89E-04 |
| nirS | 31 | 30 | 40 | 36 | 30 | 31 | 7.43E-05 | 7.41E-05 | 6.84E-05 | 6.40E-05 | 6.78E-05 | 6.31E-05 |
| Nitrite + Reduced azurin + H+ <=> Nitric oxide + H2O + Oxidized azurin | | | | | | | | | | | | |
| norB | 76 | 77 | 128 | 129 | 74 | 66 | 1.82E-04 | 1.90E-04 | 2.19E-04 | 2.29E-04 | 1.67E-04 | 1.34E-04 |
| norC | 0 | 0 | 0 | 0 | 0 | 0 | 0.00E+00 | 0.00E+00 | 0.00E+00 | 0.00E+00 | 0.00E+00 | 0.00E+00 |
| 2 Nitric oxide + 2 Ferrocytochrome c + 2 H+ <=> Nitrous oxide + 2 Ferricytochrome c + H2O | | | | | | | | | | | | |
| nosZ | 80 | 77 | 89 | 96 | 50 | 60 | 1.92E-04 | 1.90E-04 | 1.52E-04 | 1.71E-04 | 1.13E-04 | 1.22E-04 |
| Nitrous oxide + 2 Ferrocytochrome c + 2 H+ <=> Nitrogen + 2 Ferricytochrome c + H2O | | | | | | | | | | | | |

## Table S3 Relative abundance of dominant (> 0.1%) genus in refuses samples (genes/total of each sample > 1.0%, were listed)

| **Genus** | **A1** | **A2** | **B1** | **B2** | **C1** | **C2** |
| --- | --- | --- | --- | --- | --- | --- |
| Hyphomicrobium | 2.48E-03 | 3.28E-03 | 6.40E-02 | 6.02E-02 | 2.07E-02 | 2.09E-02 |
| Sphingomonas | 6.26E-02 | 5.47E-02 | 8.11E-03 | 6.81E-03 | 3.29E-03 | 3.14E-03 |
| Anaerolinea | 6.62E-03 | 7.08E-03 | 2.57E-02 | 1.13E-02 | 5.21E-02 | 2.92E-02 |
| Gemmatimonas | 3.56E-02 | 3.07E-02 | 6.83E-03 | 2.31E-02 | 7.74E-03 | 1.87E-02 |
| Anaerolineae | 1.44E-02 | 1.60E-02 | 0.00E+00 | 2.52E-02 | 0.00E+00 | 3.53E-02 |
| Ignavibacterium | 2.54E-02 | 2.75E-02 | 1.35E-02 | 1.36E-02 | 2.34E-02 | 1.79E-02 |
| Brevundimonas | 1.38E-03 | 1.84E-03 | 2.30E-02 | 2.13E-02 | 1.17E-03 | 1.28E-03 |
| Microgenomates | 3.68E-03 | 3.53E-03 | 1.44E-02 | 1.19E-02 | 1.98E-02 | 1.46E-02 |
| Pyrinomonas | 1.94E-02 | 1.54E-02 | 7.93E-03 | 5.47E-03 | 1.08E-02 | 7.86E-03 |
| Pseudomonas | 4.63E-03 | 4.57E-03 | 1.93E-02 | 1.55E-02 | 9.69E-03 | 7.29E-03 |
| Draconibacterium | 7.54E-04 | 7.47E-04 | 1.82E-02 | 1.57E-02 | 1.39E-02 | 8.52E-03 |
| Hydrogenophaga | 6.44E-04 | 5.98E-04 | 7.91E-03 | 7.27E-03 | 1.76E-02 | 1.45E-02 |
| Caldilinea | 6.12E-03 | 6.13E-03 | 1.19E-02 | 6.79E-03 | 1.74E-02 | 1.15E-02 |
| Gemmatirosa | 1.69E-02 | 1.32E-02 | 1.39E-02 | 8.66E-03 | 1.62E-02 | 1.03E-02 |
| Longilinea | 4.12E-03 | 4.46E-03 | 0.00E+00 | 6.98E-03 | 0.00E+00 | 1.50E-02 |
| Hassallia | 5.69E-03 | 6.44E-03 | 8.04E-04 | 7.57E-04 | 1.50E-02 | 1.27E-02 |
| Rhizobium | 2.33E-03 | 2.64E-03 | 1.37E-02 | 1.26E-02 | 3.36E-03 | 6.15E-03 |
| Levilinea | 4.00E-03 | 4.52E-03 | 0.00E+00 | 6.54E-03 | 0.00E+00 | 1.35E-02 |
| Bellilinea | 4.46E-03 | 4.60E-03 | 0.00E+00 | 7.34E-03 | 0.00E+00 | 1.33E-02 |
| **Shannon-Wiener index*** | 7.046674 | 7.131693 | 7.135987 | 7.122794 | 7.125873 | 7.040526 |

*** Shanno-Wiener index was calculated based on the genus with abundance > 0.1%**

## Table S4 Relative abundance of potential denitrifiers of interests in refuses samples (genes/total of each sample)

| **Genus** | **A1** | **A2** | **B1** | **B2** | **C1** | **C2** |
| --- | --- | --- | --- | --- | --- | --- |
| Anaerolinea | 6.62E-03 | 7.08E-03 | 2.57E-02 | 1.13E-02 | 5.21E-02 | 2.92E-02 |
| Anaeromyxobacter | 2.21E-03 | 2.27E-03 | 2.86E-03 | 2.03E-03 | 3.03E-03 | 2.49E-03 |
| Arenibacter | 3.51E-03 | 3.61E-03 | 1.07E-03 | 1.31E-03 | 6.64E-04 | 5.63E-04 |
| Azospirillum | 1.28E-03 | 1.40E-03 | 1.67E-03 | 1.39E-03 | 2.67E-03 | 2.08E-03 |
| Bradyrhizobium | 1.02E-02 | 1.22E-02 | 9.14E-03 | 6.79E-03 | 8.76E-03 | 7.47E-03 |
| Caldilinea | 6.12E-03 | 6.13E-03 | 1.19E-02 | 6.79E-03 | 1.74E-02 | 1.15E-02 |
| Caldithrix | 1.12E-03 | 1.34E-03 | 1.43E-03 | 1.21E-03 | 1.68E-03 | 1.25E-03 |
| Cystobacter | 1.39E-03 | 1.49E-03 | 1.30E-03 | 1.22E-03 | 2.02E-03 | 1.88E-03 |
| Desulfotignum | 2.09E-04 | 1.83E-04 | 2.53E-04 | 2.14E-04 | 3.36E-04 | 2.44E-04 |
| Fulvivirga | 2.33E-03 | 2.62E-03 | 1.19E-03 | 8.75E-04 | 5.31E-03 | 3.98E-03 |
| Hyphomicrobium | 2.48E-03 | 3.28E-03 | 6.40E-02 | 6.02E-02 | 2.07E-02 | 2.09E-02 |
| Nitrospira | 1.05E-02 | 1.09E-02 | 5.00E-03 | 8.35E-03 | 1.13E-03 | 2.66E-03 |
| Pseudomonas | 4.63E-03 | 4.57E-03 | 1.93E-02 | 1.55E-02 | 9.69E-03 | 7.29E-03 |
| Rhodanobacter | 1.32E-03 | 1.09E-03 | 8.62E-04 | 7.40E-04 | 8.42E-04 | 6.04E-04 |
| Rhodopirellula | 2.45E-03 | 2.84E-03 | 3.04E-03 | 2.71E-03 | 2.97E-03 | 2.94E-03 |
| Rhodothermus | 1.88E-03 | 1.88E-03 | 7.22E-03 | 5.61E-03 | 2.97E-03 | 2.38E-03 |
| Sorangium | 2.29E-03 | 5.72E-03 | 2.42E-03 | 1.51E-03 | 1.23E-02 | 8.82E-03 |
| Sphingomonas | 6.26E-02 | 5.47E-02 | 8.11E-03 | 6.81E-03 | 3.29E-03 | 3.14E-03 |
| Thauera | 2.34E-03 | 1.95E-03 | 1.58E-03 | 1.20E-03 | 1.23E-03 | 9.87E-04 |
| Verrucomicrobium | 4.78E-04 | 5.81E-04 | 5.39E-04 | 6.09E-04 | 9.87E-04 | 1.03E-03 |

## Table S5 Explanatory results of variance partitioning analysis (VPA)

| **Section 1** | **Explanatory** | | | |
| --- | --- | --- | --- | --- |
|  | Functional | | Genes (name) | |
| **X1** | Genes encoding nitrite reducases | | *nirS; nirK* | |
| **X2** | Genes encoding NO reducases | | *norB* | |
| **X3** | Genes encoding N*2*O reducases | | *nosZ* | |
| **Section 2** | **Partition** | | | |
|  | Default residual square | | Adjusted residual square | Testable |
| **X1** | 0.68814 | | 0.61017 | True |
| **X2** | 0.98357 | | 0.97946 | True |
| **X3** | 0.63269 | | 0.54086 | True |
| ***Section 3** | **Individual fraction** | | | |
|  | Test | Control | Explanatory portion | Testable |
| **Frac1** | **X1** | X2 + X3 | 0.00128 | True |
| **Frac2** | **X2** | X1 + X2 | 0.35761 | True |
| **Frac3** | **X3** | X2 + X3 | 0.00052 | True |

*Three factors were selected as previously described.[^1^](#_ENREF_1)


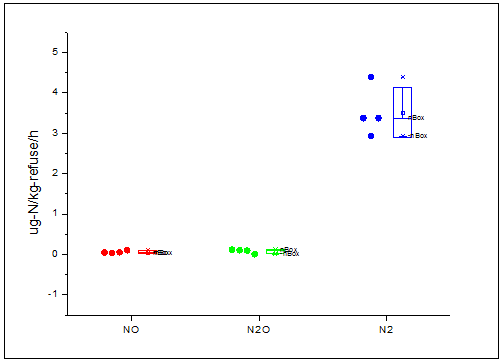


## Figure S1 The NO_x_ and N_2_ emission fluxes of raw landfill refuses (control) with no antibiotics addition (n = 4);


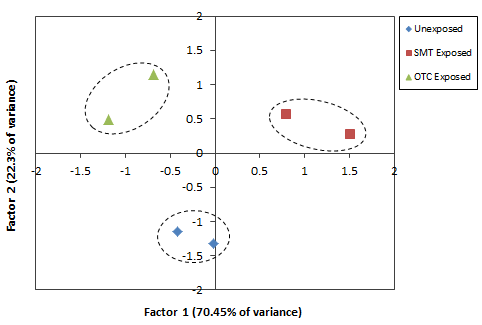


## Figure S2 Distribution of weighting factors for denitrification functional genes; Factor 1 mainly consists of *Nap, Nar* and *NorB* genes and 2 is primarily comprised of *Nir* and *NosZ* from a Principles Components Analysis (PCA) of sequenced denitrification functional genes in landfill refuses samples. Shifts in the primary component between comp. 1 and comp. 2 clusters are evident with clear disparity of gene abundances among 6 samples. The extraction and analysis methods were based on previous study: Varimax with Kaiser normalization for rotation and converged in 5 iterations; factors with the sum explained > 85 % of variance were selected for visualization.[^2^](#_ENREF_2)


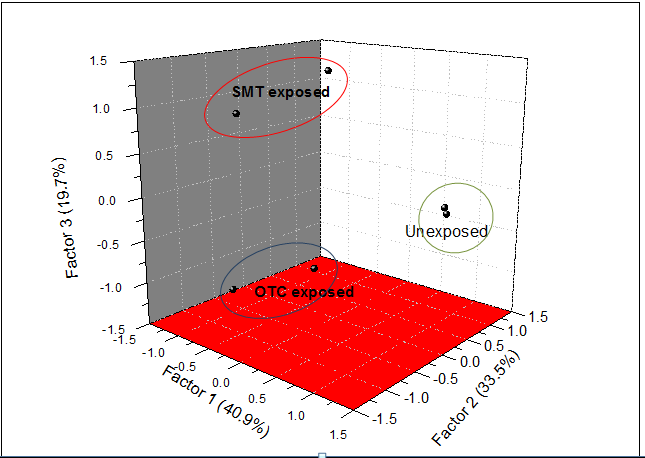


## Figure S3 Distribution of weighting factors for microbial community (genus > 0.1% of total in each sample); Shifts in the primary component between comp. 1 comp. 2 and comp. 3 clusters are evident with variation of bacteria composition (abundance) among 6 samples. The extraction and analysis methods were based on previous study: Varimax with Kaiser normalization for rotation and converged in 5 iterations; factors with the sum explained > 85 % of variance were selected for visualization.[^2^](#_ENREF_2)


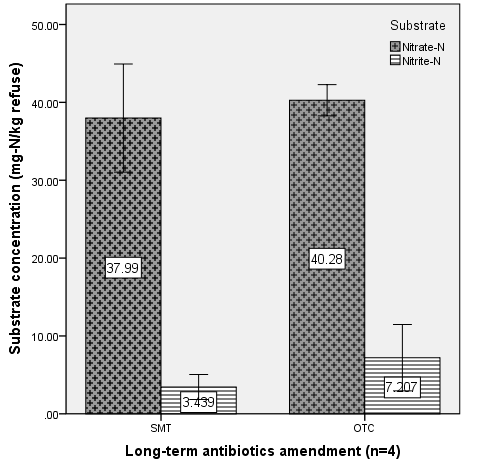


## Figure S4 Concentration of nitrate and nitrite in soil extracts after long-term antibiotics amendments;

The 24 hours emission of N was calculated < 1.0μg-N per kilogram refuse (**Fig. 1**). The sum of these emitted N and nitrite/nitrate-N residuals in amended sample, despite felling in the range of, is less than the total amount of added N (80 – 90 mg-N/kg-refuse). This might be explained by the landfill soils adsorption or loss of N in soil extraction (supporting information SI-2) for nitrate/nitrite tests. Although N-mass balance is not the foci of this study, a more precisely operated experiment including all N-species could better our understanding of antibiotic-stressed denitrification in the future.


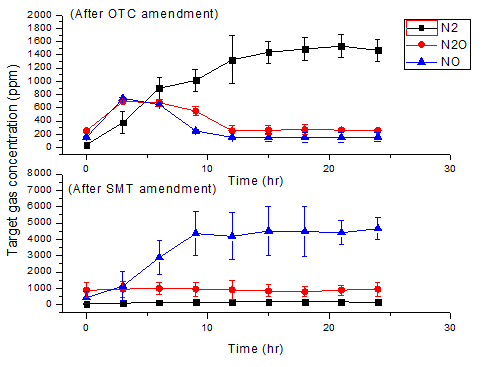


## Figure S5 The real-time target gases concentrations in 24 hours; the time “0: is defined as the time of first sampling;

Our experiments were NOT set to monitor the variation of NO_x_ & N_2_ within 24hr (though this is surely very interesting and worth further exploring) BUT to show how antibiotics and ARGs affect nitrogen gases emission in a long term (as title reads), as well as a comparison to a short term (shock) test. As plotted in Fig. S5, in the last 10-12 hours, the concentration of target gases became stable. We reckon this may indicate the denitrification process is either at a plateau or bottleneck (e.g. presumably lack of substrate or deactivation of related functional genes/enzymes). This is the reason why we chose 24 hours as the period of gas detection rather than 12 or 36hrs.

# SI-2 Extraction and analysis for leachate and refuse samples

The pretreatment of landfill leachate was optimized according to previous study. After the filtration through 0.45μm, 0.2 g Na_2_EDTA was added in 100 mL raw leachate and ultrasonicated (for Na_2_EDTA dissolve) to reduce the effects of metal cations that could potentially complex antibiotics. The obtained leachate samples were added with H_2_SO_4_ (1/10) until pH reaching 3.0.

The pretreatment of landfill refuses was based on previous research.[^5^](#_ENREF_5) 5.0 g of each freeze-dried sediment samples were weighted into a 50 mL glass centrifugal tube, followed by addition of 100 mL of the work solutions of the seven internal standards (50 ng each). Then the samples were mixed and placed in dark a refrigerator at 4℃ overnight. 20 mL acetonitrile and critic acid buffer (pH 3) solution mixture (1:1) were added into each centrifugal tube; and vortexed for 2 min. All glass tubes were then ultrasonicated for 15 min and centrifuged at 1000g for 10 min. The supernatant from each tube was removed into 200 mL glass round-bottom flask. This process was repeated twice and all resulting extracts were combined. sample was evaporated at 55℃ to remove the organic solvent, and diluted to 100 mL with ultra-pure water and pH was adjusted to 3.0 (same to the leachate volume prior to (SPE)). For the measurement of nitrate and nitrite residual in samples, 20mL KCl (1 M) solution were added. The mixture was shaken for 12 hours and then centrifuged at 3000r/min for 5 minutes. The supernates were used for following nitrite and nitrate measurement.

The clean-up and solid phase extraction (SPE) was conducted under following protocols: SAX anions exchange cartridges (CNW, 200 mg) and Oasis HLB cartridges (Waters, 500 mg) that were preconditioned by 10 mL ultrapure water and 10 mL methanol (HPLC-grade) were connected in tandem for in the cleanup of anions and extraction of antibiotics in leachate samples respectively, and the pretreated solutions were passed through the cartridges at a flow rate of 3 – 5 mL/min (Mediwax, 12). After this, the SAX cartridge was removed and the HLB cartridge was rinsed with 10 mL ultrapure water to remove weakly bound impurities and Na_2_EDTA; and vacuum dried for 1 h. All cartridges were then eluted with 3 mL × 3 ethanol (HPLC-grade) with a flow rate of 1- 3 mL/min, and the filtrate were collected in a 10 mL glass tube. The methanol eluate was dried under a gentle nitrogen stream at 45 ℃ till nearly dry and subsequently redissolved in 1 mL of methanol (HPLC-grade). The methanol solutions (filtrated through 0.22 μm membrane) were transferred to 2 mL amber vials and stored under -20℃ before analysis by UPLC-MS/MS.

For quantification, internal standard methods were utilized where sulfamethoxazole-D_4_ and demeclocycline were spiked as the internal standards in the final elute. The standard solutions (sulfamethazine (SMT) and oxytetracycline (OTC)) diluted from 0.5, 1, 5, 10, 25 to 50 ng/mL were adopted to draw linear calibration curves. After that, antibiotics contents were obtained by calculating the ratios of target analytes peak area to that of the internal standards. The limit of detection (LOD) and the limit of quantification (LOQ) were defined as signal-to-noise (S/N) of 3 and 10. The recoveries of target compounds (500 ng/L) spiked to the filtrate were between 62.1% and 129.4% (specific results were listed in **Table S1**).

Mass spectrometric (MS) analysis was conducted on a Waters triple quadrupole tandem mass spectrometer with a Z-spray electrospray interface. Both positive (SAs, FQs, TCs, MLs) and negative ion (CPs) mode was applied in the determination of the antibiotics with the following parameters: capillary voltage, 3.0 kV; cone voltage, 6-100 V; source temperature, 150 °C; desolvation gas temperature, 500 °C; desolvation gas flow, 800 L/h nitrogen; cone gas flow, 150 L/h nitrogen; argon collision gas flow, 0.17 mL/min for MS/MS. The specific operational conditions and results were all listed in the **Table S1**.

## Table S6 Antibiotics contents and matrix effects (%) of target antibiotic in refuses samples.

| **Antibiotics** | **LOD** | **LOQ** | **Matrix Effects (%)** | **Fragmenting (V)** | **CE (eV)** | **Parent ions** | **Daughter ion** |
| --- | --- | --- | --- | --- | --- | --- | --- |
| OTC | 0.05 | 0.19 | 84.8±6.02 | 119 | 17 | 461.2 | 426.1 |
|  |  |  |  | 119 | 41 | 461.2 | 201.1 |
| SMT | 0.07 | 0.45 | 110.0±12.6 | 124 | 21 | 279.3 | 186 |
|  |  |  |  | 124 | 21 | 279.3 | 124.1 |
| **Antibiotics** | **^1^Refuse samples (concentration in final elutes)** | | | | | **^2^Media** | **^2^Average** |
| OTC (ng/mL) | 4.87 | 12.1 | 5.97 | 3.25 | 2.28 |  |  |
|  | 11.4 | 9.87 | 27.9 | 13.8 | 28.3 |  |  |
|  | 8.3 | 13 | 31.4 | 2.83 | 3.37 | 0.24 (mg/kg) | 0.244 (mg/kg) |
|  | 9.34 | 14.1 | 10.1 | 9.14 | 35.3 |  | 0.18 (SD) |
|  | 9.14 | 14 | 10.5 | 12.8 | 12.6 |  |  |
| SMT (ng/mL) | 2.64 | 1.47 | 13.5 | 6.11 | 8.46 |  |  |
|  | 2.68 | 1.88 | 10.5 | 4.74 | 2.71 |  |  |
|  | 2.93 | 8.23 | 7.67 | 4.52 | 2.16 | 0.16 (mg/kg) | 0.13 (mg/kg) |
|  | 2.46 | 7.6 | 11.2 | 7.88 | 5.98 |  | 0.07 (SD) |
|  | 8.42 | 11.1 | 13.1 | 12.3 | 7.24 |  |  |

**^1^** 32 samples were collected from Shanghai Laogang Lanfill; The background antibiotic content (mg/kg-soil) = concentration/5

**SI-3 Quality control and bioinformatics**

*Quality control and assembly:* the obtained PE150bp data were firstly quality controlled, (a) removal of adaptor contaminated reads: if a read with ≥ 5bp contaminated by adaptor, the paired end reads were removed; (b) the duplicates shared 100% similarity in length and sequence were determined and removed; (c) the reads were trimmed using sickle (version 1.33) to remove low quality base pairs with the parameters of -q 20 and -l20. The quality reads were assembled using IDBA_UD software (version 1.1.1; Peng *et al*., 2012) with a range of k-mer length(20, 40, 60, 80, 100), with other parameters set as, -pre_correction. The information with respect to quality of resulting reads were listed in **Table S2**.

*Gene prediction and functional annotation*. Protein-coding genes and corresponding protein sequences were predicted using Genemark with default parameters.[^6^](#_ENREF_6) For function annotation, the protein sequences were compared against the NCBI-nr, KEGG and COG STRING databases, using BLASTp with a threshold of e-value≤10-5 (**Table S2**).

*Functional categories*. For functional categories analyses of the protein-coding genes, including COG, COG category, KO, and KEGG pathway, the functional annotation of them were parsed, and the relative abundance of genes assigned to each functional category was calculated with the consideration of its corresponding coverage, for example, the calculation of the relative abundance of COG0001.

$$\text{COG0001 \% }=\text{ }\frac{\sum_{\text{i }=\text{ 1}}^{\text{n}} \text{a}_{\text{i}}\text{C}}{\sum_{\text{j}=1}^{\text{m}} \text{b}_{\text{j}}\text{C}}\text{ }\times\text{ 100\%}$$

where a_i_C represents the coverage of gene a assigned as COG0001, b_j_C represents the coverage of any gene assigned as a COG.

## Table S7 The reads details of de novo assembly and gene prediction

| **Contigs** | | **Refuse samples** | | | | | |
| --- | --- | --- | --- | --- | --- | --- | --- |
|  |  | **A1** | **A2** | **B1** | **B2** | **C1** | **C2** |
| Numbers | | 288541 | 282925 | 394435 | 376429 | 308648 | 341585 |
| Total length（bp) | | 202935949 | 195142653 | 298194995 | 290887629 | 216734527 | 242560244 |
| Max. length（bp） | | 98622 | 103960 | 312095 | 380526 | 330321 | 258031 |
| Mean length（bp） | | 703 | 690 | 756 | 773 | 702 | 710 |
| N50（bp） | | 765 | 737 | 842 | 886 | 765 | 780 |
| Mean GC（%） | | 59.02 | 59.00 | 57.77 | 56.98 | 57.78 | 58.39 |
| **Genes** | **Refuses samples** | | | | | | |
|  | **A1** | | **A2** | **B1** | **B2** | **C1** | **C2** |
| Numbers | 417385 | | 404959 | 584726 | 562414 | 442586 | 491185 |
| Total length（bp) | 176855730 | | 168861156 | 257066604 | 251538108 | 187716591 | 210529992 |
| Max. length（bp） | 9225 | | 9225 | 11823 | 11316 | 12786 | 17772 |
| Mean length（bp） | 424 | | 417 | 440 | 447 | 424 | 429 |
| Mean GC（%） | 59.64 | | 59.65 | 58.57 | 57.75 | 58.42 | 59.00 |

# References

1 Zhang, Y. *et al.* High Concentrations of the Antibiotic Spiramycin in Wastewater Lead to High Abundance of Ammonia-Oxidizing Archaea in Nitrifying Populations. *Environ Sci Technol* **49**, 9124-9132, doi:10.1021/acs.est.5b01293 (2015).

2 Graham, D. *et al.* Antibiotic Resistance Gene Abundances Associated with Waste Discharges to the Almendares River near Havana, Cuba. *Environ. Sci. Technol.* **45**, 418-424, doi:10.1021/es102473z (2011).

3 Wu, D., Huang, Z., Yang, K., Graham, D. & Xie, B. Relationships between antibiotics and antibiotic resistance gene levels in municipal solid waste leachates in Shanghai, China. *Environ Sci Technol* **49**, 4122-4128, doi:10.1021/es506081z (2015).

4 Yan, C. *et al.* Antibiotics in the surface water of the Yangtze Estuary: occurrence, distribution and risk assessment. *Environmental Pollution* **175**, 22-29, doi:10.1016/j.envpol.2012.12.008 (2013).

5 Zhou, L. J. *et al.* Trends in the occurrence of human and veterinary antibiotics in the sediments of the Yellow River, Hai River and Liao River in northern China. *Environmental Pollution* **159**, 1877-1885, doi:10.1016/j.envpol.2011.03.034 (2011).

6 Peng, Y., Leung, H. C., Yiu, S. M. & Chin, F. Y. IDBA-UD: a de novo assembler for single-cell and metagenomic sequencing data with highly uneven depth. *Bioinformatics* **28**, 1420-1428, doi:10.1093/bioinformatics/bts174 (2012).
